# Phosphohistone H3 and Ki‐67 as prognostic markers in metastatic small intestinal neuroendocrine tumours: A comparative, retrospective cohort study

**DOI:** 10.1111/jne.70188

**Published:** 2026-05-10

**Authors:** Dimitrios Papantoniou, Malin Grönberg, Eva Tiensuu Janson

**Affiliations:** ^1^ Department of Medical Sciences, Endocrine Oncology Uppsala University Uppsala Sweden; ^2^ Department of Oncology Ryhov County Hospital Jönköping Sweden

**Keywords:** Ki‐67, PHH3, phosphohistone H3, siNET, small intestinal neuroendocrine tumours

## Abstract

Ki‐67 index and mitotic count form the basis of grading of small intestinal neuroendocrine tumours (siNET). We hypothesized that the mitosis‐specific marker phosphohistone H3 (PHH3) might better correlate with cancer‐specific survival (CSS) and with response to treatment. We evaluated the association between Ki‐67 index, PHH3‐estimated mitotic count, and survival outcomes in a retrospective cohort of 73 consecutive patients with metastatic siNET. Additionally, we estimated the optimal cut‐off for PHH3 and cross‐validated the outcome. Both markers adequately distinguished CCS when comparing lower and higher proliferation groups (Ki‐67: 128 vs. 95 m; PHH3: 149 vs. 88 m). They were strongly associated with CSS as continuous (HR 1.18 [1.08–1.28] and 1.16 [1.09–1.25]), and dichotomous variables (HR 2.96 [1.31–6.67] and 3.11 [1.50–6.46]). The Cox model based on PHH3 displayed slightly better optimism‐corrected Harrell's c‐index (0.71 vs. 0.68) and Akaike information criterion (219 vs. 223). Additionally, PHH3 showed significant association with PFS after treatment with somatostatin analogues (HR 1.12 [1.03–1.21]), and borderline significant association with PFS after treatment with peptide receptor radionuclide therapy (HR 1.11 [1.00–1.24]). A cut‐off of >2 mitoses per 10 high‐power fields estimated by PHH3 seemed to have better discrimination power compared to the standard WHO cut‐off (<2). Mitotic count based on PHH3 is associated with CSS and with PFS after treatment with first‐line SSA and possibly with PRRT for metastatic siNET. It may be an alternative to Ki‐67 for estimation of proliferation and grading. A cut‐off of >2 mitoses per 10 HPF might better distinguish G1 and G2 tumours.

## 1 INTRODUCTION

Neuroendocrine tumours of the small intestine (siNET) are rare tumours, with an incidence of 1.05–1.46 per 100,000 persons.^1^ They are often slow‐growing tumours and might be associated with long survival even when metastasized. According to the World Health Organization (WHO), tumour cell proliferation rates form the basis of siNET classification into three grades.^2^ Grade 1 tumours are characterized by cell proliferation index Ki‐67 <3% and <2 mitoses per 2 mm^2^, or traditionally 10 high power fields (HPF). They are generally indolent, slowly progressing tumours and tend to respond well to treatment with somatostatin analogues (SSA).

Grade 2 siNET have a slightly higher Ki‐67 index (3%–20%) and/or mitotic rate (2–20 per 2 mm^2^) and might also respond to SSA and everolimus, at least in the lower proliferation range. Grade 3 siNET are much more infrequent tumours exhibiting Ki‐67 >20% and/or >20 mitoses per 10 HPF. They tend to progress quickly, and the effect of standard treatments is uncertain in this category group.

Ki‐67 is an antibody binding to a chromatic protein expressed in nuclei in G1, S, G2 and M phases during cell proliferation. As it is absent during the G0 phase of the cell cycle, it has become a widely used, sensitive marker of cell proliferation.^3^ Ki‐67 index is calculated in slides with immunohistochemical staining for Ki‐67 by counting between 500 to >2000 cells and determining the percentage of Ki‐67 positive cells. This is typically a time‐consuming process, reported to require up to 10 min per case.^3^ Moreover, although Ki‐67 might be prognostic of outcomes after treatment with SSA, its association with efficacy of peptide‐receptor radionuclide therapy (PRRT) is less clear.^4^ Mitoses are counted in routine haematoxylin‐eosin (H&E) slides. The major drawbacks are heterogeneity, interobserver variability and the risk for non‐specific staining of apoptotic, necrotic or crashed cells and of other non‐proliferating cells undergoing repair of inflammation.^5^

Phosphohistone‐H3 (PHH3) is a protein related to chromatin structure. It is a histone H3 isoform which becomes phosphorylated during late G2 and M phases of the cell cycle and could thus allow for easier identification of mitoses.^6^ In contrast to Ki‐67, PHH3 specifically labels cells in the late phases of cell division. Additionally, it does not label cells undergoing apoptosis.^7^ It has thus been investigated as a marker of mitotic activity in neuroendocrine tumours of mostly pancreatic and lung origins.^3^, ^8^, ^9^, ^10^, ^11^, ^12^, ^13^, ^14^, ^15^, ^16^, ^17^ However, it has not been specifically evaluated in siNET.

We hypothesized that PHH3 would be a better prognostic marker for cancer‐specific survival (CSS) and response to treatment in patients with siNET in comparison with Ki‐67, and that the mitotic cut‐off proposed by WHO for H&E slides might not be applicable to the mitotic count as estimated by PHH3.

## 2 MATERIALS AND METHODS

### 2.1 Patients

For this retrospective cohort study, all 73 patients with metastatic inoperable siNET with available formalin‐fixed, paraffin‐embedded (FFPE) tumour blocks were extracted from a previously published internal database.^4^ In short, patients with siNET, characterized as G2 at either the original or subsequent biopsies, and who were treated between 2000 and 2019 at Uppsala University Hospital, were eligible for inclusion. Approximately 30% of the patients had G1 tumours in the original biopsy used for the purpose of this study. The study was approved by the Uppsala ethical review board. Survival status was censored on 31 October 2023, or at last known contact. Patients dying from causes unrelated to their NET tumour were censored at the time of death.

### 2.2 Histology

In this study, immunohistochemical stainings were conducted on FFPE sections using fully automated protocols (DAKO Autostainer Link48) and the Envision FLEX, high pH detection kit from DAKO #K8000. The antibodies used included rabbit polyclonal for PHH3 (Sigma Aldrich, #06‐570, diluted 1:300), mouse monoclonal for Chromogranin A (CgA, ThermoFisher, #MA5‐13096, clone LK2H10, diluted 1:600), and mouse monoclonal for Ki‐67 (Dako, #M7240, clone MIB‐1, diluted 1:50). For Ki‐67 detection, the standard protocol was modified by using Low pH Target Retrieval Solution (Dako, #K8005). Positive controls for PHH3 and Ki‐67 (human tonsil tissue) and for CgA (human pancreas tissue) were used.

### 2.3 Proliferation index and mitotic index

CgA‐stained slides were reviewed. D.P., M.G. and E.T.J. independently analysed Ki‐67 and the PHH3‐estimated mitotic index. First, the region with the highest labelling (hotspot) was identified in Ki‐67‐stained slides under low magnification. In high magnification, at least 500 tumour‐cell nuclei were analysed. The proliferation index was estimated as the ratio of positive Ki‐67 nuclei to the total number of nuclei. The PHH3 mitotic index was counted as the total number of tumour cells with prominent staining in 10 consecutive high‐power fields (HPF, x400 magnification) at the area of higher proliferation.

## 3 STATISTICAL ANALYSIS

CSS was calculated from the date of metastatic disease to cancer‐related death, and progression‐free survival (PFS) from the day of treatment start to disease progression. Survival outcomes were analysed using the Kaplan–Meier method, and between‐group differences were evaluated using a log‐rank test. Hazard ratios (HR) and confidence intervals (CI) were estimated from unadjusted Cox proportional hazards models. The predictive ability of Ki‐67 index and PHH3 mitotic count was compared with Harrell's c‐index (higher is better) and with Akaike information criterion (AIC, lower is better).^18^ Optimal cut‐off points were calculated with R packages Survminer 0.4.3 and maxstat 0.7–25 using the maximally selected rank statistics, a method that allows the evaluation of cut‐off points, which provide the classification of different risk groups in a quantitative or ordered predictor variable.^19^ A cross‐validation was performed by randomly splitting the sample into training and testing dataset, and repeating the operation 1000 times. A frequency distribution of the optimal cut‐offs was then graphically presented.

As an additional analysis, improvements in risk prediction were evaluated using the continuous Net Reclassification Improvement (NRI) at 3 and 5 years. The NRI quantifies how well a new model correctly reclassifies individuals into higher predicted risk categories (for those who experience events) and into lower risk categories (for those who did not), compared with a reference model. Reclassification was assessed for models including PHH3 alone and for the combination of PHH3 and Ki‐67, relative to Ki‐67 alone. Continuous NRI and 95% confidence intervals were computed using the nricens R package, which estimates predicted risks directly from the fitted Cox models and accounts for censoring via the Kaplan–Meier method with 1000 bootstrap iterations, as described by Pencina et al.^20^

Statistical analysis was performed with R (R Foundation for Statistical Computing, Vienna, Austria). *p* values <.05 were considered statistically significant.

## 4 RESULTS

### 4.1 Demographics

Seventy‐three patients with available pathology specimens were included in this study. Of those, 56% were male. Median age at diagnosis was 62 years (interquartile range, IQR, 57–69). Material from the primary tumour was used in 40 cases (55%), from liver biopsies in 28 cases (38%), and from other locations in 5 cases (7%). Baseline patient characteristics are summarized in Table 1.

**TABLE 1:** Baseline patient characteristics.

	*N* = 73
Sex, *n* (%)	
Female	32 (44%)
Male	41 (56%)
Age at diagnosis, median (IQR)	62 (57–69)
Biopsy location, *n* (%)	
Liver	28 (38%)
Primary	40 (55%)
Other	5 (7%)
Ki‐67, median (IQR)	4 (2–8)
PHH3, median (IQR)	2 (0–5)
Grade, Ki‐67, *n* (%)	
G1	23 (32%)
G2	50 (68%)
Grade, PHH3, *n* (%)	
G1	35 (48%)
G2	38 (52%)

*Note*: Values are median (interquartile range) or *n* (%).

Abbreviations: G1, Grade 1; G2, Grade 2; PHH3, phosphohistone H3.

**FIGURE 1:**
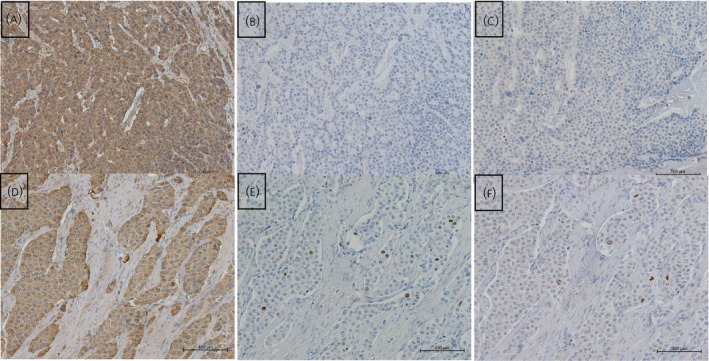
Grade 1 and 2 small intestinal neuroendocrine tumours in slides stained for Chromogranin A (A and D), Ki‐67 (B and E) and phosphohistone H3 (C and F).

**FIGURE 2:**
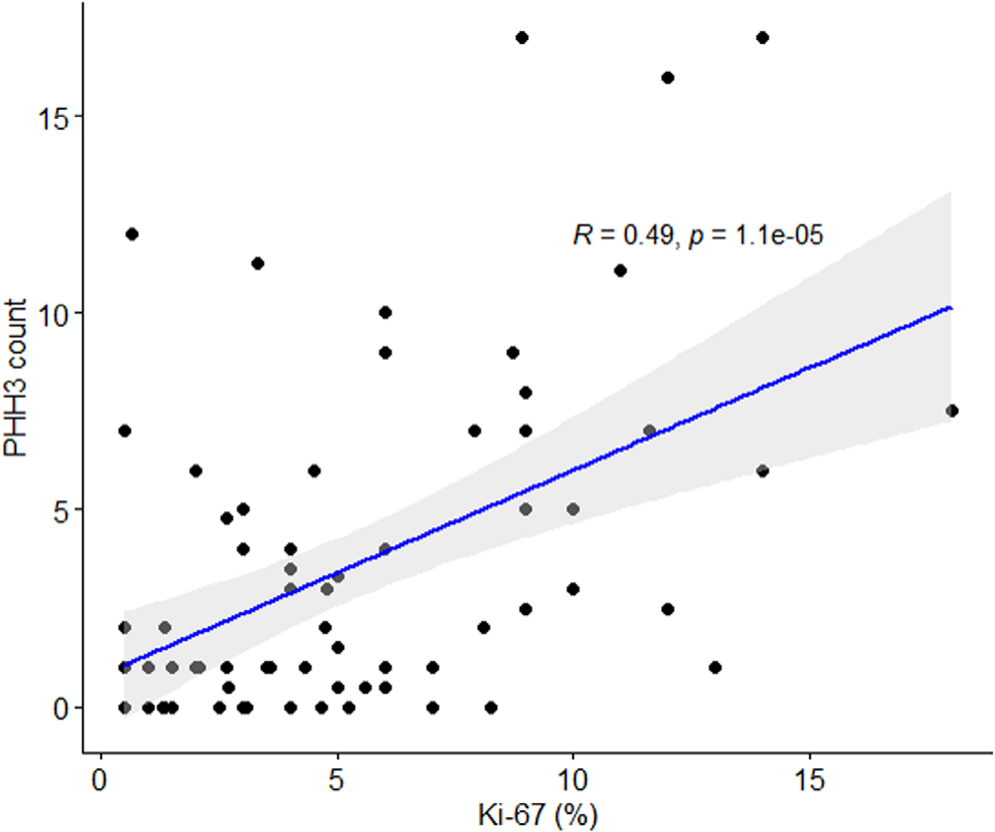
Correlation between PHH3 and Ki‐67.

Representative examples of CgA, Ki‐67, and PHH3 staining are shown in Figure 1. Median Ki‐67 index was 4% (IQR 2–8), and median PHH3‐estimated mitotic count was 2 per 10 HPF (IQR 0–5). Based on Ki‐67, 68% of patients had Grade 2 tumours, whereas based on mitotic count, as measured by PHH3, 52% had G2 tumours. Pearson's correlation between Ki‐67 and PHH3 was 0.49 (Figure 2).

### 4.2 Association with disease outcomes

**FIGURE 3:**
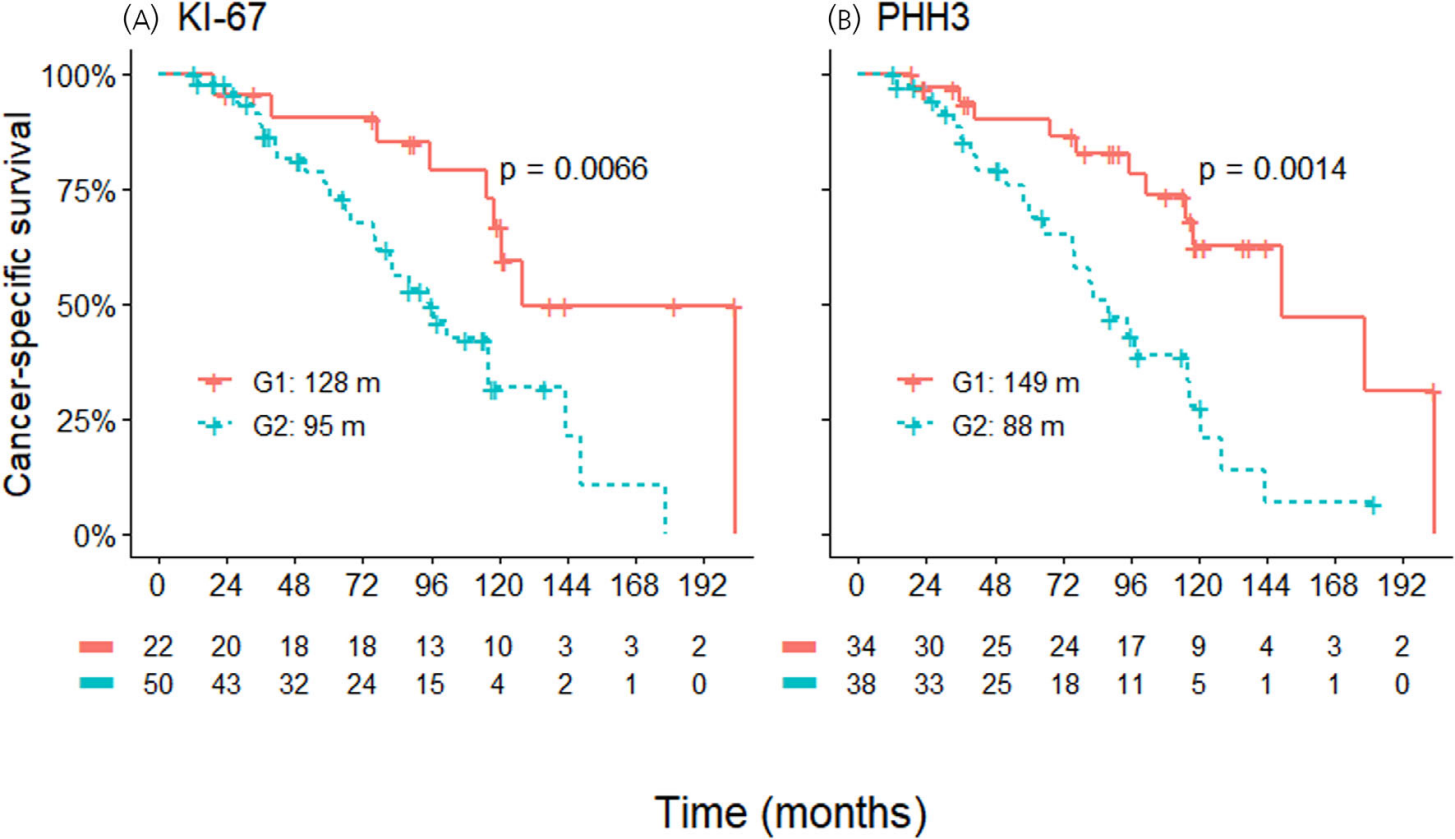
Cancer‐specific survival by grade, as evaluated by (A) Ki‐67, (B) phosphohistone H3 (PHH3). Both proliferation markers can adequately separate patients in groups of better and worse prognosis. G1, Grade 1; G2, Grade 2; m, months.

Tumour grade, as estimated by both Ki‐67 and by PHH3, was significantly associated with CSS (Figure 3). In the case of Ki‐67, Grade 1 tumours had a median CSS of 128 months compared to 95 months for Grade 2 tumours (*p* = .007). For PHH3, G1 tumours had a median CSS of 149 months, and G2 tumours of 88 months (*p* = .001).

We further investigated the relationship between Ki‐67 and PHH3 with CSS using an unadjusted Cox regression model. Both variables were significantly associated with survival, with HR of 1.18 (1.08–1.28) and 1.16 (1.09–1.25), respectively. In the Ki‐67 model, the optimism‐corrected Harrell's c‐index was 0.68, whereas in the PHH3 model, it was 0.71. A higher c‐index indicates a better model. We additionally compared AIC between the two models. A lower AIC indicates a model which better fits the data. The results still favored PHH3 as the marker with the better predictive ability (223 respectively 219, Table 2).

**TABLE 2:** Predictive values of Ki‐67 and PHH3 for CSS.

Marker	HR (95% CI)	Grade 2 vs. 1, HR (95% CI)	c‐index	AIC
Ki‐67	1.18 (1.08–1.28)	2.96 (1.31–6.67)	0.68	223
PHH3	1.16 (1.09–1.25)	3.11 (1.50–6.46)	0.71	219

*Note*: A semi‐parametric cox regression model was used to evaluate Ki‐67 and PHH3 as predictors of CSS. HR and CI were comparable for the markers examined as continuous variables and dichotomous variables. The models were compared with Harrell's c‐index (higher is better), and AIC (lower is better). Metrics slightly favoured the PHH3 model.

Abbreviations: AIC, Akaike information criterion; CI, confidence intervals; CSS, cancer‐specific survival; G1, Grade 1; G2, Grade 2; HR, hazard ratios; PHH3, phosphohistone H3.

### 4.3 Association with response to treatment

Fifty‐two patients received treatment with first‐line somatostatin analogues (SSA). Of those, 26 patients were later treated with peptide‐receptor radionuclide therapy (PRRT). Only PHH3 was significantly associated with PFS after SSA (HR 1.12 [1.03–1.21]), whereas the association for Ki‐67 was not significant (HR 1.08 [0.99–1.17]). A similar trend was observed in patients treated with PRRT (HR for PHH3 1.11 [1.00–1.24] and for Ki‐67 0.93 [0.82–1.07]).

### 4.4 Optimal cut‐offs for PHH3

**FIGURE 4:**
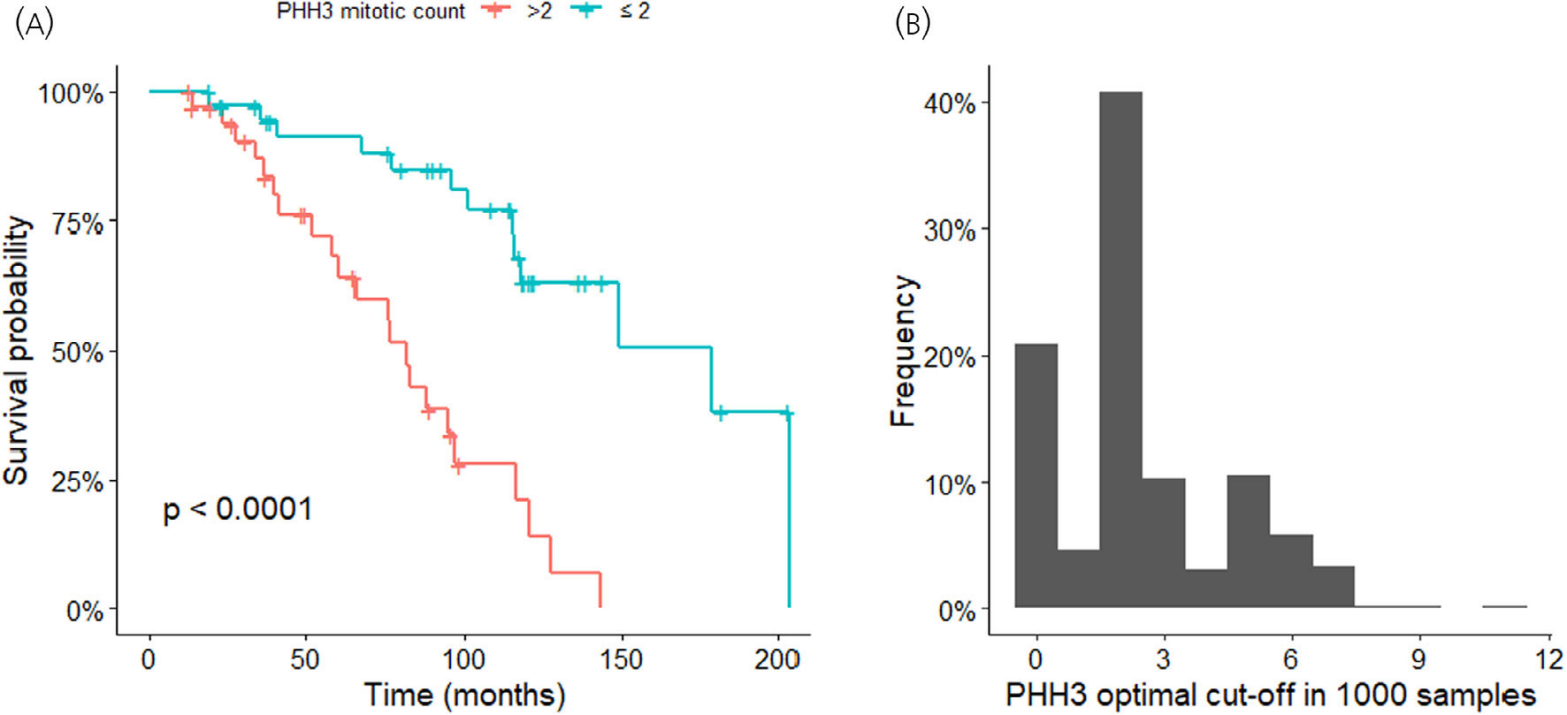
The most usual optimal cut‐off for the phosphohistone H3 (PHH3)‐estimated mitotic count was >2 in 1000 samples. Using a cut‐off of >2 mitoses per 10 high‐power fields (HPF) provided slightly better separation of survival curves.

As the mitoses cut‐offs in WHO classification refer to mitoses counting on H&E stains, these might not be optimal for staining with PHH3. We estimated the optimal cut‐off for PHH3 to be >2 mitoses per 10 HPF. This cut‐off provided a better separation of patients in lower and higher risk categories, compared to the usual cut‐off (179 m vs. 82 m, *p* < .0001, Figure 4A).

In order to internally validate the procedure, we split the sample equally in training and testing dataset. We repeated the process 1000 times, and created a histogram of the distribution of estimated optimal cut‐offs (Figure 4B). Two mitoses per 10 HPF was the most frequent cut‐off. Finally, we estimated HR in the test samples. The median HR was 4.99 for G2 vs. G1 tumours, as estimated by the new cut‐off. It was significant in 95% of the samples. Based on this data, we suggest a cut‐off of >2 mitoses per 10 HPF, as counted on PHH3 stain, for patients with siNET.

### 4.5 Net reclassification improvement

At 3 years, replacing Ki‐67 with PHH3 in a Cox model for CSS resulted in a modest improvement in risk reclassification (continuous NRI 0.40 [95% CI –1.11 to 1.23]), suggesting some patients might be more appropriately assigned to higher or lower risk categories. However, no improvement was observed at 5 years (NRI –0.03 [−0.99 to 0.84]). In an additional explorative step, we examined the effect of combining both PHH3 and Ki‐67. The resulting model showed a more pronounced reclassification gain at 3 years (NRI 0.53 [−0.48 to 1.41]) and a slightly smaller improvement at 5 years (NRI = 0.36 [−0.22 to 1.06]). None of the estimates reached statistical significance.

## 5 DISCUSSION

In this study of patients with metastatic siNET, we showed that PHH3 is associated with cancer‐specific survival. Compared to Ki‐67, PHH3 seemed to be better associated with PFS after treatment with SSA, and possibly with PRRT. A cut‐off of >2 was the most usual cut‐off value chosen after cross‐validation, and discriminated well between better and worse prognostic groups.

Ki‐67 index is the marker most widely used in pathology in order to evaluate cell proliferation. It has been associated with prognosis, and possibly with response to treatment.^4^ Assessment of Ki‐67 might be done through ‘eyeballing’, manual counting on slides or printed images, or with automated methods, and is generally time‐consuming. ‘Eye‐balling’ is the fastest method, but it is associated with interobserver variability and is not recommended.^21^ Manual counting in hotspots is the gold standard and is recommended by the WHO. However, it is a time‐intensive method as it requires counting of at least 500 and up to 2000 cells, and can be affected by tumour heterogeneity, hotspot selection and field size. For example, a recent study in pancreatic NET showed that using a smaller field size was associated with a higher Ki‐67 index estimation.^3^ These issues have led to an increasing interest for automatic systems; such methods have yielded mixed results, with some studies reporting poor concordance with manual analysis of Ki‐67,^22^ whereas others suggest that digital pathology methods could replace manual counting.^10^

WHO additionally recommends counting mitoses on standard H&E‐stained slides. However, apoptotic cells might be mistaken for mitoses, and the process is challenging even for experienced pathologists. As PHH3 is a protein predominantly expressed during the M phase, it facilitates the recognition of cells undergoing mitosis. A study of pancreatic NET reported almost perfect interobserver agreement (intraclass correlation coefficient *k* ≥ 0.98), and the time required for recognition of mitoses was significantly shorter (3.67 vs. 1.68 min for 50 HPF, *p* < .0001).^16^ Mitotic counts based on PHH3 have been reported to be higher compared to those based on H&E slides, and generally grading based on PHH3 with a standard WHO cut‐off would result on a higher grade compared to routine WHO grading.^9^, ^16^

In our study, grading by Ki‐67 recognized more tumours as G2, compared with grading by PHH3. Both grading systems were strongly associated with CSS (Figure 3). When examined as continuous variables in a Cox model, PHH3 seemed to better discriminate survival outcomes, based on a higher Harrell's c‐index and a lower AIC (Table 2).

Assessing the proliferation rate of the tumour by Ki‐67 provides prognostic information, but its association with treatment outcomes remains unclear. We have previously shown that a higher Ki‐67 index is associated with treatment outcomes in Grade 2 siNET treated with SSA but not with PRRT.^4^ On the contrary, in the current study there was a borderline association between PHH3‐estimated mitotic count and PFS in PRRT‐treated patients. As PHH3 is highly specific for mitoses, whereas Ki‐67 additionally stains G1, S and G2 phases, PHH3 might better represent tumour biology and proliferation rate, and thus be better associated with treatment outcomes.

Both Ki‐67 and PHH3 are continuous variables. Even if dichotomizing a continuous variable might result in loss of prognostic power, it is common praxis to categorize tumours in distinct grade groups for the purpose of patient counselling and potentially treatment selection. WHO suggests a cut‐off value of 3% for Ki‐67, and of <2 mitoses per 10 HPF counted on H&E slides, to distinguish grade 1 and grade 2 tumours. Previous studies conducted in pancreatic NET have suggested optimal cut‐offs of 2,^12^ 4,^16^ 7^15^ and 10^13^ to maximize discrimination in groups of better and worse prognosis. However, siNET tend to have lower proliferation indexes and mitoses counts compared to pancreatic NET, and estimations derived from the latter might not apply in siNET. In our study, a cut‐off of >2 mitoses per 10 HPF seemed to offer the best discrimination.

In an additional exploratory analysis, PHH3 showed modest improvement over Ki‐67 in reclassifying the risk of cancer‐specific death, with this effect appearing more pronounced when it was combined with Ki‐67. However, the wide confidence intervals crossing zero, likely reflecting the limited number of events, warrant cautious interpretation of these findings.

We acknowledge that the 73 siNET cases included in our study are a relatively small sample. Additionally, PFS data were available for only two thirds of the patients treated with SSA and one third of those treated with PRRT. Our findings should thus be validated in a larger cohort. Most importantly, an automated system for assessment of Ki‐67 and PHH3 could be evaluated in a future study.

In conclusion, PHH3 might be an alternative to Ki‐67 for the grading of metastatic siNET tumours and correlates with both CSS and with PFS after first‐line SSA and possibly PRRT. Based on our data, we suggest a cut‐off of >2 mitoses per 10 HPF based on PHH3 to better distinguish between G1 and G2 tumours.

## AUTHOR CONTRIBUTIONS

**Dimitrios Papantoniou:** Conceptualization; investigation; methodology; writing – review and editing; visualization; formal analysis; writing – original draft; data curation. **Malin Grönberg:** Investigation; writing – review and editing; methodology; resources. **Eva Tiensuu Janson:** Funding acquisition; writing – review and editing; supervision; resources; conceptualization; investigation.

## FUNDING INFORMATION

This study was supported by the Swedish Cancer Society (20 0921) and Futurum – the Academy for Health and Care, Region Jönköping County.

## CONFLICT OF INTEREST STATEMENT

Dimitrios Papantoniou reports a relationship with Camurus AB that includes: consulting or advisory.

## ETHICS STATEMENT

The study was approved by the Uppsala ethical review board (EPN 2017‐403). All procedures were performed in accordance with the 1964 Helsinki Declaration and its later amendments. Patients provided written consent at the time of treatment for the use of tumour samples and clinical data in retrospective research projects. The ethical review board waived the requirement for renewal of patient consent.

## Data Availability

The data that support the findings of this study are available upon reasonable request from the corresponding author (D.P.). The data are not publicly available due to their containing information that could compromise the privacy of research participants.

## References

[1] Lamarca A , Bartsch DK , Caplin M , et al. European neuroendocrine tumor society (ENETS) 2024 guidance paper for the management of well‐differentiated small intestine neuroendocrine tumours. J Neuroendocrinol. 2024;36(9):e13423. doi:10.1111/jne.13423 38977327

[2] Klimstra D , Kloppell G , La Rosa S , Rindi G . Classification of neuroendocrine neoplasms of the digestive system. WHO Classification of Tumours: Digestive System Tumours. 5th ed. WHO Classification of Tumours Editorial Board (Ed), International Agency for Research on Cancer; 2019:111.

[3] Huang W , Nebiolo C , Esbona K , Hu R , Lloyd R . Ki67 index and mitotic count: correlation and variables affecting the accuracy of the quantification in endocrine/neuroendocrine tumors. Ann Diagn Pathol. 2020;48:151586. doi:10.1016/j.anndiagpath.2020.151586 32836178

[4] Papantoniou D , Grönberg M , Thiis‐Evensen E , et al. Treatment efficacy in a metastatic small intestinal neuroendocrine tumour grade 2 cohort. Endocr Relat Cancer. 2023;30(3):e220316. doi:10.1530/ERC-22-0316 36629395 PMC9986391

[5] Thunnissen FBJM , Ambergen AW , Koss M , Travis WD , O'Leary TJ , Ellis IO . Mitotic counting in surgical pathology: sampling bias, heterogeneity and statistical uncertainty. Histopathology. 2001;39(1):1‐8. doi:10.1046/j.1365-2559.2001.01187.x 11454038

[6] Hendzel MJ , Wei Y , Mancini MA , et al. Mitosis‐specific phosphorylation of histone H3 initiates primarily within pericentromeric heterochromatin during G2 and spreads in an ordered fashion coincident with mitotic chromosome condensation. Chromosoma. 1997;106(6):348‐360. doi:10.1007/s004120050256 9362543

[7] Hendzel MJ , Nishioka WK , Raymond Y , Allis CD , Bazett‐Jones DP , Th'ng JPH . Chromatin condensation is not associated with apoptosis*. J Biol Chem. 1998;273(38):24470‐24478. doi:10.1074/jbc.273.38.24470 9733739

[8] Fung AD , Cohen C , Kavuri S , Lawson D , Gao X , Reid MD . Phosphohistone H3 and Ki‐67 labeling indices in cytologic specimens from well‐differentiated neuroendocrine tumors of the gastrointestinal tract and pancreas: a comparative analysis using automated image cytometry. Acta Cytol. 2013;57(5):501‐508. doi:10.1159/000351475 24021213

[9] Kim MJ , Kwon MJ , Kang HS , et al. Identification of Phosphohistone H3 cutoff values corresponding to original WHO grades but distinguishable in well‐differentiated gastrointestinal neuroendocrine tumors. Biomed Res Int. 2018;2018:1‐10. doi:10.1155/2018/1013640 PMC5892266 29780816

[10] Lea D , Gudlaugsson EG , Skaland I , Lillesand M , Søreide K , Søreide JA . Digital image analysis of the proliferation markers Ki67 and Phosphohistone H3 in Gastroenteropancreatic neuroendocrine neoplasms: accuracy of grading compared with routine manual hot spot evaluation of the Ki67 index. Appl Immunohistochem Mol Morphol. 2021;29(7):499‐505. doi:10.1097/PAI.0000000000000934 33758143 PMC8354564

[11] Mathian É , Drouet Y , Sexton‐Oates A , et al. Assessment of the current and emerging criteria for the histopathological classification of lung neuroendocrine tumours in the lungNENomics project. ESMO Open. 2024;9(6):103591. doi:10.1016/j.esmoop.2024.103591 38878324 PMC11233924

[12] Ozturk Sari S , Taskin OC , Gundogdu G , et al. The impact of phosphohistone‐H3‐assisted mitotic count and Ki67 score in the determination of tumor grade and prediction of distant metastasis in well‐differentiated pancreatic neuroendocrine tumors. Endocr Pathol. 2016;27(2):162‐170. doi:10.1007/s12022-016-9424-9 26936845

[13] Tracht J , Zhang K , Peker D . Grading and prognostication of neuroendocrine tumors of the pancreas: a comparison study of Ki67 and PHH3. J Histochem Cytochem. 2017;65(7):399‐405. doi:10.1369/0022155417708186 28651471 PMC5490847

[14] Tsuta K , Liu DC , Kalhor N , Wistuba II , Moran CA . Using the mitosis‐specific marker anti–phosphohistone H3 to assess mitosis in pulmonary neuroendocrine carcinomas. Am J Clin Pathol. 2011;136(2):252‐259. doi:10.1309/AJCPDXFOPXGEF0RP 21757598

[15] Villani V , Mahadevan KK , Ligorio M , et al. Phosphorylated histone H3 (PHH3) is a superior proliferation marker for prognosis of pancreatic neuroendocrine tumors. Ann Surg Oncol. 2016;23(5):609‐617. doi:10.1245/s10434-016-5171-x 27020585 PMC8713440

[16] Voss SM , Riley MP , Lokhandwala PM , Wang M , Yang Z . Mitotic count by Phosphohistone H3 Immunohistochemical staining predicts survival and improves Interobserver reproducibility in well‐differentiated neuroendocrine tumors of the pancreas. Am J Surg Pathol. 2015;39(1):13‐24. doi:10.1097/PAS.0000000000000341 25353284

[17] Zhao CL , Dabiri B , Hanna I , et al. Improving fine needle aspiration to predict the tumor biological aggressiveness in pancreatic neuroendocrine tumors using Ki‐67 proliferation index, phosphorylated histone H3 (PHH3), and BCL‐2. Ann Diagn Pathol. 2023;65:152149. doi:10.1016/j.anndiagpath.2023.152149 37119647

[18] Burnham KP , Anderson DR . Multimodel inference: understanding AIC and BIC in model selection. Sociol Methods Res. 2004;33(2):261‐304. doi:10.1177/0049124104268644

[19] Hothorn T , Lausen B . Maximally Selected Rank Statistics in R. Accessed 29 February 2020 https://cran.r‐project.org/web/packages/maxstat/vignettes/maxstat.pdf

[20] Pencina MJ , D'Agostino RB , D'Agostino RB , Vasan RS . Evaluating the added predictive ability of a new marker: from area under the ROC curve to reclassification and beyond. Stat Med. 2008;27(2):157‐172; discussion 207–212. doi:10.1002/sim.2929 17569110

[21] Reid MD , Bagci P , Ohike N , et al. Calculation of the Ki67 index in pancreatic neuroendocrine tumors: a comparative analysis of four counting methodologies. Mod Pathol. 2015;28(5):686‐694. doi:10.1038/modpathol.2014.156 25412850 PMC4460192

[22] Hacking SM , Sajjan S , Lee L , et al. Potential pitfalls in diagnostic digital image analysis: experience with Ki‐67 and PHH3 in gastrointestinal neuroendocrine tumors. Pathol Res Pract. 2020;216(3):152753. doi:10.1016/j.prp.2019.152753 31761497

